# The Role of Food Antioxidants, Benefits of Functional Foods, and Influence of Feeding Habits on the Health of the Older Person: An Overview

**DOI:** 10.3390/antiox6040081

**Published:** 2017-10-28

**Authors:** Douglas W. Wilson, Paul Nash, Harpal Singh Buttar, Keith Griffiths, Ram Singh, Fabien De Meester, Rie Horiuchi, Toru Takahashi

**Affiliations:** 1 School Medicine Pharmacy and Health, Durham University, Durham TS17 6BH, UK; 2 Centre for Ageing & Dementia Research, Swansea University, Swansea SA2 8PP, UK; P.Nash@swansea.ac.uk; 3 Department of Pathology & Laboratory Medicine, Faculty of Medicine, University of Ottawa, Ottawa, ON K1H 8M5, Canada; hsbuttar@bell.net; 4 Tenovus Institute for Cancer Research, Cardiff University, Laurel Cottage, Castleton, Gwent CF3 2UR, UK; profkgriffiths@aol.com; 5 Halberg Hospital and Research Institute, Civil Lines, Moradabad, UP 244001, India; rbs@tsimtsoum.net; 6 TsimTsoum Institute, Ulica Gołębia 2, Kraków 31-007, Poland; fdm@tsimtsoum.net; 7 Department of Food Sciences and Nutrition, Faculty of Human Environmental Sciences, Mukogawa Women’s University, 6-46 Ikebiraki-cho, Nishinomiya 663-8558, Japan; mhor9496@mukogawa-u.ac.jp; 8 Graduate School of Human Environment Science, Fukuoka Women’s University, 1-1-1 Kasumigaoka, Higashi-ku, Fukuoka 813-8529, Japan; takahashi@fwu.ac.jp

**Keywords:** older person, diet, food flavor/color/aroma, olfactory function, dementia, Alzheimer’s disease, food antioxidants, human ecology, food industry

## Abstract

This overview was directed towards understanding the relationship of brain functions with dietary choices mainly by older humans. This included food color, flavor, and aroma, as they relate to dietary sufficiency or the association of antioxidants with neurodegenerative diseases such as dementia and Alzheimer’s disease. Impairment of olfactory and gustatory function in relation to these diseases was also explored. The role of functional foods was considered as a potential treatment of dementia and Alzheimer’s disease through inhibition of acetylcholinesterase as well as similar treatments based on herbs, spices and antioxidants therein. The importance of antioxidants for maintaining the physiological functions of liver, kidney, digestive system, and prevention of cardiovascular diseases and cancer has also been highlighted. Detailed discussion was focused on health promotion of the older person through the frequency and patterns of dietary intake, and a human ecology framework to estimate adverse risk factors for health. Finally, the role of the food industry, mass media, and apps were explored for today’s new older person generation.

## 1. Introduction

This section outlines the approach to this overview with some definitions and caveats associated with the title.

### 1.1. Health

Logically, it seemed reasonable that the integration of neural, cellular and humoral variations of their components that constitute temporal aspects of physiology manifested in body rhythms in good health are indeed a “consensus partium in tempore” (consensus of parts in time) [1], which suggests that the characterization and quantification of body rhythms were different in health and disease among the young or the older person, and males or females. Such age and gender differences may allow the identification/determination of rhythmic changes that mark the progression of a healthy subject to one with occult and overt abnormalities.

### 1.2. Older Person

We classify this sub-heading as being part of the process of senescence (natural ageing) or senility (pre-disease or during disease) [2], though temporal changes between the two states is often difficult to establish because of the experiment design which often focuses on the duration of study and required outcomes and confounding factors such as the inclusion/exclusion characteristics of the recruits to clinical trials (e.g., Body Mass Index (BMI), health definitions, birth cohort, etc.).

### 1.3. Food Antioxidants

Antioxidants, natural or synthetic food preservatives, are additives that preserve food from “farm to plate” and militate against oxidative deterioration on storage and processing. Due to their high stability and low volatility, the antioxidants help to maintain the level of nutrients, the texture, color, taste, freshness, functionality, aroma, and appeal to consumers such as the older person, ceteris paribus. Antioxidants [3] are not only in food additives but are also to be found in food supplements and levels should be measured, as such, in body tissues and fluids [4]. Lesser known sources of antioxidants to that cited in reference [3] abound, for example, black chokeberry (*Aronia melanocarpa*) found in juices, purees, jams, and so forth which, containing high levels of polyphenols and flavonoids, has potential interventive value for a range of chronic diseases such as diabetes and cardiovascular diseases [5]. Fermented grain food supplements also contain antioxidants, e.g., Antioxidant Biofactor, reducing lipid oxidation by scavenging upon the peroxyl radical [6].

Antioxidants are essential for animal and plant life since they are involved in complex metabolic and signaling mechanisms. They protect plants by producing phytoalexins, e.g., isoflavonoid structures, in response to microbiological and fungal pathogen invasion [7]. In terms of dietary intervention, mechanisms relating to, for example, microglia senescence and neural ageing should be sought which allow effective nutrient treatments to be developed in the form of e.g., functional foods and plant supplements that may develop our understanding of abnormal ageing and onset of neurodegenerative diseases [8].

Excellent reviews (>100; 2015–2017 Medline searches and other sources) on free radicals and related topics which impact on human health e.g., Lobo et al. [9], However the blind should not lead the blind [10] because for many reasons due to study design, insufficient statistical power, the nature of cohorts, the follow-up timespan, response evaluation, etc., conflicting results may arise and affect health promotion programs e.g., resveratrol [11]. An increasingly active area of research is the existing metabolome of the human gut either under the influence of a “usual” diet or one which may be transformed or manipulated by dietary antioxidant interventions, e.g., flavonoids, carotenoids, etc. focusing on gut-brain interactions, vide infra. This overview focused on the health of older persons, of which neurodegenerative diseases are becoming more prevalent, Increasingly, as more structural information is gleaned from natural products possessing antioxidative, anti-inflammatory, anti-amyloidogenic properties, new synthetic structures can be developed to potentially become the next generation of product for these diseases, such as Alzheimer’s disease: as reported by Hiremathad [12,13].

In simplest terms q.v. [3], food antioxidants are scavengers of “free” (an unnecessary term) radicals, which is a chemical structure that has at least one unpaired electron which can cause cellular and genetic changes due to their highly reactive state that can act to produce damage over the nm range, e.g., the hydroxyl (HO•) radical; other oxygen radicals include the hydroperoxyl (HOO•), alkyloxyl (ROO•), and superoxide anion (O_2_^−^•); an important nitrogen containing radical is nitric oxide (NO•); sulfur containing radicals include thiyls (RS•) and disulfide anions (RSSR^−^•); and carbon containing radicals include the carbonate (CO_3_^−^•) group [14]. A detailed review about the radicals and food antioxidants can be found in a paper from this group (Griffiths et al.) [3].

As would be expected, of the four million radicals produced per day, a balance must be struck between potential cellular/tissue damage and the benefits of radicals needed for healthy intra- and inter-cellular signaling processes. Indeed, the oxidation of food as a source of body heat and locomotion was due to Lavoisier in 1775; an analogy being fuel for a power-driven vehicle; and reported some years later [15].

### 1.4. Feeding/Food

This term refers to that which is eaten. It also embodies actions associated with the timing and frequency of meal times; the role of caregivers [16] and the patient’s low dietary/nutrient intake [16] such as knowing when to “give in” or “step in” with the patient at meal-time [17] especially in hospitals and nursing institutions [17], with restricted dietary intake and potential beneficial changes to longevity [18,19]. Such actions are conducted to optimally add “years to life” and “life to years”, give way to perhaps perceptions of dietary composition and health, with benefits to physical and mental health outcomes that are the underlying topic of this communication. Prima facie: it is reasonable to explore whether biological/chronological ageing [2] is significantly associated with reduced function or malfunction of vital organs, e.g., heart and kidney [20] and changes in microbiota [21] and digestive enzymes, and transporters for the absorption of food products, micronutrients (minerals, vitamins, etc.) from the gastrointestinal tract; as well as drug-food/herbal interactions which may take place between the gut and liver cytochrome-450 isozymes and poly-glycoprotein transporters (PGPs).

In the context of feeding evolution, the literature abounds with observations on birds, bats, butterflies, even bears, and other animals, such as rats, consuming the juices from fermented fruits or indeed nectar from hymenoptera originating in the Triassic Period. Presumably, this practice of feeding on fermented food materials took place before the dawn of mankind when, even today, hawks prey upon their impaired smaller cousins and birds espy and consume the beguiled insects. In an evolutionary context of antioxidants, perhaps 50 million years before the Great Oxygen Event perhaps some 2.3 billion years ago [22], atmospheric oxygen became increasingly present, though variable, in the once-anaerobic world and defense mechanisms were evolved to combat its toxicity and adapt to the developing world up to the falling level of 21% of today. “Circadian” periods were also increasing, Williams [23], due to tidal friction and there was an increase in the diversity of life forms with eukaryotes in post-Precambrian times which had longer lifespans and were able to develop their energy needs beyond a single day-length. The distribution of oxygen occurrence in the atmosphere, oceans, and land, became relatively stable and free radicals, oxidants, antioxidants were essential for the survival of life forms, and as such are important today from the metabolic, wholesome food and human medicine perspectives.

### 1.5. Food and Agriculture-Evolution and Refashioning of Food Supply for Health: Past to Future

Wider knowledge of food harvesting/gleaning, storage, preservation, practices, and cultures may enhance our potential for healthy eating. Even in Britain in the 1950s food produce was limited by availability, cost and tradition compared to today. Turning the clock back, as Steinkraus (1993) [24] stated, an interaction between man, microbe, and food existed in Paleolithic times [25], some 10,000 years ago before more elaborate farming took place i.e., the time of transition from hunting and gathering with “plant breeding” programs in Neolithic times. Neolithic crops developed as part of an important advancement in early agriculture (i.e., wild progenitors to emmer wheat, einkorn, barley, flax, chick pea, pea, lentil, bitter vetch), and species of domesticated animals, viz. cows, buffaloes, goats, sheep, and pigs that were no longer lean (change in meat composition) by running from pursuing hunters. Much can be learned from the dietary development of those in the Fertile Crescent i.e., [26] the Cradle of Civilization, those in Levant in the South Eastern part of Turkey, and Crete, and the emergence of a so-called “Mediterranean diet”, and that of a Roman diet of bread, wine, and from past integration of produce and practices from the Middle Ages with a strong influence from Islamic culture.

Today’s “Mediterranean diet” comprises vegetables, fruits, seeds, nuts, beans, cereal grains, and starchy food e.g., bread or pasta, olive oil and fish. N-3 fatty acids, components of the Mediterranean diet, affect brain function particularly in neurogenesis and neuroplasticity and have a role to play in cognition, mood, and behavior [27]. The relatively high intake of plant-derived antioxidant foods compared to Northern European and American diets is striking as is the longevity of their inhabitants, e.g., Cretan. It is South America, though not exclusively, with its abundant and diverse plant life that can make a contribution towards anti-inflammatory and healthy foods. It was 35 Ma during late Eocene that South America [28] was isolated from other geostructures originating further back to Gondwanaland and even earlier to the super continent of Pangaea. Land colonization by plants, probably from marine algae, increased the paradoxical life-giving oxygen that could also harm plants and they developed elaborate phyto-signaling mechanisms involving anti-oxidants which also potentially damaged the plant itself: though perhaps not as much as previously thought [29]. Phytoalexins developed to protect plants, as part of a short-term response, often in the form of reactive oxygen species, to an invading pathogen. Early domestication of plants took place associated with many crops containing antioxidants, preceded by neuroplasticity and pre-human evolution under very different climatic conditions e.g., maize in South America to pre-Colombian human settlements [30,31] in the neotropical regions in the early Holocene epoch of Peru and Mexico from evidence based on phytoliths, and starch grains on grinding stones.

Climate change threatens food security and alternative crops/foods for human consumption need to be found in lands affected by drought, e.g., tree pods of the genus *Prosopis* garnered by pre-Columbian humans e.g., in the ABC countries in the form of flour, syrup, alcoholic beverages [32] and latterly fed to animals because of competitive European crops in the market. These foods have significant antioxidant content as would be expected in abiotic stress. Clearly, human survival-age has increased over time and its role in intrinsic causes of death, antagonistic pleiotrophy or programmed death is not yet fully understood. Adequate food, calorific intake, is essential but restricted calorific consumption may increase longevity through weight loss by timing of dietary intake from the Natick studies of Halberg’s group [33].

## 2. Factors Related to Feeding of the Older Person

The impact of dietary nutrients on the health of persons of all ages is complex and multifactorial, and chemosensory, involving biology, food antioxidants, chronobiology, environment, culture, religion, eating habits, memory loss, intake of natural products and herbal remedies (such as phytoalexins, polyphenols, carotenoids, spices and aromatic herbs, alcoholic and non-alcoholic beverages), commercial and marketing hype, language, interventions (pharmacological and “non-drug”), special cuisines, nursing and domestic care (intravenous and tube feeding), primary nutrients like omega-3 fatty acids, (e.g., alpha-linolenic acid) carbohydrates (glucose-monosaccharide-energy), amino acids (tryptophan), vitamins (B12, B6, C) and trace elements. From the biosystems perspective and intervention, it is evident from many clinical trials and meta-analysis lead to the reverse engineering concept of the erstwhile healthy individual’s part of the heart-brain- mind-gut axes as well as their components (e.g., membranes, cerebral vasculature neurones, and oligodendrocytes), astrocytes and metabolomics/ microbiota. These biosystems are important in ameliorating disease onset or progression. From the patient care perspective, several factors (appetite, food appeal, disease severity, tube feeding, social interaction) are important as is the health of the caretaker. Some of these factors are discussed below.

Clearly, food must be appealing to the older person in presentation, texture, color [34,35], taste, flavor and aroma [36] provided olfactory function is not impaired [37]. Non-traditional food/herbs may bestow benefits [38]: even then: do the phytochemicals have the same effect when given alone or in combinations with other foodstuff [39]? Flavor comprises the perception of bitterness and sweetness in the mouth. However, dangers exist in “reward” centers in the brain, e.g., obesity and metabolic syndrome [40]; though hyperphagia or hunger in patients with dementia, a process that is complex, leads to possible over- or under-eating. Aroma volatiles of fruit, for example, are specific to species and variety. These volatiles may contain aliphatic esters e.g., strawberries [41] (DWW unpublished data, c2003), phenolic compounds (even in alcohol-free wine [42]), and sesquiterpenes, etc. [43]. In food, humans, are exposed to food interactions e.g., “Advanced Glycation End Products” (proteins/lipids exposure to sugars) [44] which are often associated with the ageing person and Alzheimer’s disease.

The palate of an older person will depend on dietary history, chronobiological age [2], disease status and severity- particularly neurodegeneration, or oesophageal cancer, or other related diseases: so an appetizing diet must take cognizance of the consumer’s, masticatory ability [45] and training thereof using mirror neurons (possibly located in the premotor cortex, supplementary motor area, primary somatosensory cortex, and inferior parietal cortex.) and the dining experience [46], even utensil-less [47], and must consider social isolationism. These facets of nutrition choices, notwithstanding socioeconomic restrictions, relating to the health of the older person are now discussed. Cellular meat, viz. cellular agriculture and dietary consumption is beyond the scope of this article; and taste matching of food components in the older person is beyond the expertise of the authors. Consideration to meal times for health improvement include changes to food service, obviously food improvement, dining environment, staff training and feeding assistance and meta-analysis of the care home situation is a useful tool to identify inconsistent outcomes [48].

### 2.1. Food Color, the Brain, and Dietary Sufficiency

Study of food color as an appealing consumer aid to ingestion and dietary sufficiency has probably 4 main threads, viz. evolution (beyond the scope of this review-area of solar energy utilization and photosynthesis; early history [49]); local and global natural regulated food dye usage that obviates the need for potentially harmful synthetic dyes [50], though not infallibly so from a safety viewpoint; and as an associated (chemical structures in their own right or as a result of chemical interaction with other constituents) functional food property with health giving properties; and culinary skills including the colors not only of the food/beverage but the color of the tableware such as plates, glasses, cups, and tablecloth evoking color contrasts which may be translated into “food” appeal/perception [51], just as we can use geometry, spatial configuration can be used to create an illusion of size of plate. Or else we may have “edible provoking” perfumes or “edible provoking” hues; perhaps these may work together or in tandem depending on neurodegeneration characteristics. Visual acuity and learning ability may be impaired in cases of deficiency of docosahexaenoic acid and arachidonic acid which are found in the membranes of the brain and retinal cells, as revealed by animal studies [52]. The role of color and other factors may be important as an interactional component within a caregiving dyad of patients with advanced dementia failing to ingest sufficient nutrients [53].

### 2.2. Food Aroma, the Brain, and Dietary Sufficiency

Chemosensory disorders of smell, apart from a warning function, may dictate our food preference and hence our food sufficiency in the older person [54] particularly in patients with Alzheimer’s disease [55]. The future aim should be to integrate nutritional needs of the older person and compensate for chemosensory deficits, through socioeconomics and innovative agricultural and food industry processes [56]. The testing for smell disorders may be cross-cultural and in a cosmopolitan community may be difficult [57].

In a review by Atterns et al. [58], olfactory dysfunction was highly prevalent in those >65 years with a rising age-trend. This review gave possible physiological causes of olfactory dysfunction and raised the possibility that it would be a useful indicator of early dementia. Of importance is the possible link between olfaction and cognition. There may be a recall of odor problem with ageing, especially with Alzheimer’s disease and Parkinson’s disease, so food appeal is probably blunted. Olfactory disorders arise also from anatamopathological neurological structural changes and may affect food appeal; and the anatomy of olfaction has been reviewed in the older person population [59]. Greater than 75% of subjects over the age of 80 have olfactory problems [60] but it is important to control for cognitive dysfunction when assessing the age effect [61,62]. Neuroimaging studies may reveal the processes associated with olfaction and inadequate dietary intake and disease progression [63].

For the aged, or even mild neurodegenerative population, stimulation of the olfactory system by aromatherapy, or multisensory stimulation using the Snoezelen method, may complement existing treatment strategies [64]. The value of aromatherapy as a treatment for dementia still remains unclear and more work is needed [65]. Cognisance should be taken of indications that in early Parkinson’s disease olfactory deficits (biomarkers) are linked to cholinergic and dopamine denervations and possibly hippocampal dysfunction and therein lies the possibility of screening for neurodegeneration [52]. As an aside, it is possible that extracellular vesicles may lead to new therapies for neurodegenerative diseases via biomarkers which echo intracellular events from regions of the Central Nervous System (CNS) and subsequent passage of biomarkers to blood and cerebrospinal fluid and may identify the cell type of origin and thence therapy.

Whereas social interaction is deliberately self-limited e.g., with those who have oral/throat cancer, when eating food, the inability to readily appreciate food, or have a distorted perception of same, from an olfactory-physiological viewpoint [36], can be measured subjectively [66], possibly brought about by Lewy body disease and damage to other parts of the brain [67]. Also tests of olfactory function may be cross-cultural. So in assessing the health of the older person, hyposmia perhaps opens the door to possible developing states of neurological problems [68]. An analogy with harmony and the sheathing of melody [69] (ninth century: now basic biochemistry) with limitation of later chords (current research) springs to mind, where olfaction may distinguish between Alzheimer’s disease and depression [70] because olfactory processes affect different parts of the brain.; so food appreciation through odor may be different: however, good longitudinal studies are needed to use olfaction testing to predict the onset of Alzheimer’s disease [71] and hence impact on feeding.

### 2.3. Food Flavor, the Brain and Dietary Sufficiency

In this section, we discuss mechanisms related to flavor rather than dietary composition (q.v. Griffiths et al. [3]; [72]) with more specific intervention action, for example, one broad (“mechanistic”) observation from a study of a variety of foods constituting a normal diet in Alzheimer’s disease subjects, gustatory (tasting) impairment was an associative agnoia in mild Alzheimer’s disease, suggesting a dissociation of olfactory and gustatory thresholds at a central level [73]. It is generally believed that the substantial neuronal brain loss, e.g., Alzheimer’s disease, affects gustatory function [74] but perhaps not in peripheral transmission of such information from taste buds (salty, bitter, sweet, umami and sour) to gustatory nerve fibers [74]. There are chemoreceptors in the human brain for olfaction and taste and their expression is different in Parkinson’s disease and dysregulation of these types of receptors in the entorhinal cortex and frontal cortex in Alzheimer’s disease and response [75] is an area in which functional foods may play a role in this pathological process.

Furthermore, it seems that there is a decline in gustatory function and smell with the severity of dementia, particularly in Parkinson’s disease [76]. It would be useful if a reliable test could be devised for identifying such differences in gustatory function, and cognitive attributes (q.v. dietary intake in dementia patients [77]) e.g., in Alzheimer’s disease, depression and a health age-controlled group of patients [78], including such parameters as taste thresholds: perhaps using additional potential diagnostic tests e.g., ApoE ε4 (apolipoprotein with allele ε4), fluorodeoxyglucose positron emission tomography, and Magnetic Resonance Imaging (MRI) appear to be useful [79]. Flavor and odor deficits have been identified in some frontotemporal lobular dementia groups; which may relate to grey matter atrophy in the anteromedial temporal lobe [80]. It has been suggested, albeit on too few subjects, that semantic dementia may demonstrate abnormal food behaviors [81].

### 2.4. Potential Role of Functional Foods for Prevention of Neurodegeneration

Acetylcholinesterase inhibitors are used to treat neurological disorders, including Alzheimer’s disease (AD), and it has been suggested that some plant-derived dietary agents like functional foods [82] may be target candidates for treating Alzheimer’s disease. Functional foods consist of natural or processed foods that contain known or unknown biologically active compounds and are effective for promoting health and wellbeing beyond dietary needs. These foods provide clinically proven and documented health benefits for the prevention, management, and treatment of chronic disease. This definition of functional foods is important as it contains key words for the candidate food, e.g., “clinically proven and not speculative”, and “documented”, indicating evidence-based trials which have been properly reported in the literature; as well as relating to intact species and not ex vivo as in cell culture.

However, much of this section is speculative from this viewpoint. For example, extracts of *Hemidesmus indicus* and *Vanilla planifolia*, contain the phenolics 2-hydroxy-4-methoxybenzaldehyde and 4-hydroxy-3-methoxybenzaldehyde which have been shown to demonstrate inhibitory potential against acetylcholinesterase and utility, notwithstanding any toxicity, for treating Alzheimer’s disease and other neurological problems [83] is possible. Butane-2,3-dione is a compound which affects amyloid-beta (1–22) by binding to the α-amino acid arginine in residue 5 and causing it to form insoluble sheet structures in cell culture [84].

Discourse follows on natural products such as fruit (raspberries), garlic, tea, which often seem to be a panacea for treating all ailments- this cannot be so. Keeping this point in mind, some functional foods and herbs appear to have medicinal potential. Diet is an essential factor that contributes to health and disease as in the normal ageing process, and reactive oxygen species that, ceteris paribus, cause inflammation and cellular damage leading to cardiovascular disease, diabetes mellitus, obesity, and Alzheimer disease [85,86,87,88]. From the onset it should be emphasized that tea, garlic, fruits, specific plants, etc. are not identical in themselves: variables include species, variety(cultivar), strain, geographic location, agricultural practice, soil type, climate, season, flush, storage, processing, transport, blending, consumer and culinary practice, etc.: even before being integrated into a meal thereafter ingested and exposed to the temporal gut microbiome. However, evidence has accrued which demonstrate the intervention potential of flavonoids, carotenoids, vitamins, herbs and spices in oxidative stress-induced diseases.

For example, red raspberries (*Rubus idaeus*) have numerous polyphenolic compounds such as ellagitannins and anthocyanins, which may reduce the risk of or reverse metabolically associated pathophysiologies [72]. Abundant experimental research has been conducted that offers some hope for future Alzheimer’s subjects e.g., the work of Professor Giulio Pasinetti [89] from Mount Sinai Hospital on orally administered grape seed extracts to rats. His group investigated the bioavailability of phenolic acids, generated by microbiota metabolism of anthocyanidins in the extracts, increasing the content of “3-hydroxybenzoic acid and 3-(3′-hydroxyphenyl)propionic acid”, resulting in the brain accumulations of the same which potently interfere with the assembly of β-amyloid peptides into neurotoxic β-amyloid aggregates that play key roles in Alzheimer’s disease pathogenesis [90].

### 2.5. Herbal Antioxidants

Garlic contains many biologically active compounds which purport to be beneficial to human health [3]; one such garlic-preparation contains the water soluble antioxidant S-allyl mercaptocysteine (aged garlic). Chauhan has reviewed the beneficial effects of garlic and its constituents on neuronal physiology and brain function; and the potential of dietary garlic as a pharmacotherapy for Alzheimer’s disease [91]. Despite these and other claims, cross-sectional studies of overall dietary patterns or specific foods (fruit and vegetables) or antioxidants or flavonoids in an older cohort using the same sample have not shown that biomarkers (C-reactive protein (CRP) and fibrinogen) of systemic inflammation relate to flavonoids or antioxidants, though a balanced or Mediterranean diet was favorable [92]: however, the vagaries of cross-sectional studies of narrow age bands is questionable.

### 2.6. Food and Antioxidants

Intervention, particularly with prodromal cognitive decline, with vitamin D which is an antioxidant and a neurosteroidal hormone with protective properties may supplement the efficacy of memantine [93]. Brain function depends on dietary nutrients, and implicit in the definition of functional foods/molecular species are target organs such as regions of the brain, with omega-3 polyunsaturated fatty acids [94], and B, E and D vitamins, etc. A review of nutritional status and brain/nervous systems and potential neuroprotection by dietary constituents is provided by Bourre [95]. There has been an explosive interest in the microbiome and the impact of dietary patterns on cognitive decline [96].

If the standardization and predictive strength of dietary patterns using functional foods to improve the health of the older person, in particular, can be realized this may be a significant advancement [97].

Studies of the immune system have demonstrated the role of antioxidants in balancing the free radical production that is needed for functional purposes against oxidative stress, which increases with age. Monitoring the immune system in response to antioxidant dietary intake may be another indicator of health or disease [98]. Aerobic exercise in humans has an increasing effect on superoxide dismutase levels and enhances the antioxidant defense mechanism yielding health benefit along with risk reduction for metabolic syndrome, diabetes and cardiovascular diseases [99], which combined with food restriction on, for example, a balanced diet, may provide extra benefit [100].

Culinary processing is a risk factor for toxicity caused by a reaction between amino acids and sugars. It typically occurs when foods with high starch content such as potatoes, root vegetables and bread, are cooked at high temperatures (over 120 °C) in a process of frying, roasting or baking. It is ameliorated by choosing plant products, low in precursors, and adding as cooking reagents which slow acrylamide formation; and reducing physical cooking conditions, etc. [101]. Risk factors for unhealthy eating also include food contaminants, depending on origin. For example, grape pomaces used in plant food supplements and food coloring may contain the mycotoxin ochratoxin A which has nephrotoxic and carcinogenic properties [102]; heavy metals [103] which in aquatic nutrient fed horticultural systems may be extracted by reeds [104], [105]; and pesticides [106,107], etc.

It is possible that in Alzheimer’s disease oxidative injury occurs in different parts of the brain via different mechanisms affecting nucleic acids, sugars, proteins, etc., so a battery of antioxidants may be advantageous including vitamin C, ubiquinone, flavonoids (e.g., tea, chocolate, etc.), carotenoids, spices and herbs, lipoic acid, melatonin and curcumin, etc., vide supra [108]. Chakraborty et al. have identified hesperidin, a flavonone glycoside, found in citrus fruit as a possible intervention agent by acting on the site of the beta amyloid precursor protein-cleaving enzyme 1 (BACE1 enzyme), producing conformational change, which inhibits amyloid fibril formation [109].

One of these compounds, viz. lipoic acid or its reduced form, being a precursor of mitochondrial enzymes, increases acetylcholine production and chelates redox-active transition metals, diminishes hydroxyl radicals, scavenges reactive oxygen species (ROS), thereby increasing the levels of reduced glutathione. The R-alpha-enantiomer, may be used to treat Alzheimer’s disease and other dementias [110]. It is noteworthy that this enantiomer occurs naturally in plants and animals.

Longitudinal studies of dietary intake in centenarians have demonstrated a low level of inflammation and satisfactory levels of antioxidants and micronutrients, e.g., zinc, copper, selenium, that have an important role in maintaining the immune system. Micronutrient-gene interactions and protein-metal speciation analysis can indicate healthy ageing and longevity [111]. Dietary studies are notoriously difficult to undertake to reveal specific unambiguous outomes. Trichopoulou’s group undertook an analysis of 850 potentially eligible studies of which 10 met the inclusion criteria and were analysed. Flavonoids, beta-carotene, and vitamin E were found to be reasonably beneficial and an adverse effect of selenium was also observed over time in global cognition [112] but the evidence is unconvincing and sparse: q.v. paper by Darlenska et al. [113].

A hitherto unmentioned role of iron in the brain has indicted neurodegenerative diseases in the older person because it has a major role in oxygen transportation and neurotransmitter production. Therapies aimed at reducing iron to decrease neurodegenerative diseases in humans may produce a beneficial outcome [114].

### 2.7. Spices and Aromatic Herbs

According to Griffiths et al. [3], “A spice is a seed, fruit, root, rhizome, bark, resin, berry, bud, stigma, or vegetable substance primarily used for seasoning and provides sweet or savory flavorings, colors or preserves food, or is a medicinal product, cosmetic or simply a vegetable”. Spices include coriander, fennel, mustard, cinnamon, mace, clove, saffron, ginger, asafetida, bay leaf oil, cinnamon, cloves, cumin, fenugreek, turmeric, poppy seed, pomegranate, red chili, sesame seed, and so forth. Many countries have spice mixtures, e.g., chaat masala (Pakistan and India). Herbs are parts of leafy green plants used for flavoring or to garnish culinary dishes. Aromatic herbs include thyme, sage, oregano, parsley, dill, marjoram, chives, rosemary, mint, lemon grass, etc. Of course, spices and herbs are used as fresh, dry, or paste preparations. It has been reported that antioxidant capacity is still present during the processed end-product, as are the total phenolics, some being less degraded than others [115], e.g., in many herbs and spices the total phenolic content remains unchanged in the processed end-product.

## 3. Selected Organs and Antioxidants

### 3.1. Hepatoprotective Dietary Antioxidants and Their Potential for Treating Alzheimer’s Disease

The eponym “Alzheimer’s disease” introduced by Kraepelin in 1910 for the disease discovered by Alois Alzheimer in 1906 [116] has long sought an effective treatment for the observed pathology described as senile plaques and neurofibrillary tangles found in the cerebral cortex of one Auguste Deter, a 51 year old woman from Frankfurt who in 1901 displayed characteristic symptoms of dementia.

Berberine, a protoberberine group of benzylisoquinoline alkaloids, found in *Berberis vulgaris* and in many other plants, and been used in Chinese medicine for over 5 Ka and is claimed to have a protective effect against a wide variety of diseases and may be a second-generation treatment for Alzheimer’s disease due to it antioxidant properties [117], and suppression of hepatic oxidative stress [118]. Caution is the watchword to avoid unsubstantiated claims, e.g., tea, that may not be evidence-based. However, it has been shown to attenuate hyperphosphorlylation and cytotoxicity induced by Calyculin A of the microtubule associated protein tau and retards neurofibrillary tangles and, vide supra, inhibits oxidative stress in Alzheimer’s disease.

Screening plants for such alkaloids may be productive, particularly if linked to consumption with little liver or other organ toxicity. Quite apart from grape seed proanthocyanidin extract, vide supra, with its high bioavailability and its protection against liver and kidney damage [119], *Silybum marianum*, which is known under a variety of names such as milk thistle; sometimes eaten in past times, is a is a medicinal plant that has long been used as a hepatoprotective remedy. It has been used for the treatment of numerous liver disorders characterized by functional impairment or degenerative necrosis. Its hepatoprotective activity is unique and acts in different ways, including antioxidant and anti-inflammatory activities, cell permeability regulator and membrane stabilizer, stimulation of liver regeneration and inhibition of deposition in collagen fibers, which may lead to cirrhosis.

Most of documented data with *Silybum marianum* are about liver disorders; however, recently several beneficial properties on a wide variety of other disorders such as renal protection, hypolipidaemic and anti-atherosclerosis activities, cardiovascular protection, prevention of insulin resistance, especially in cirrhotic patients, cancer, and Alzheimer’s disease prevention is noted, but potentially questionable due parsimony of high quality data [120,121]. A flavonoid derived from this plant, silymarin, has been identified as a possible neuroprotective agent for Parkinsonism and Alzheimer’s disease, etc., due to it antioxidative stress properties on the brain [122].

Evidence suggests that accumulations of amyloid-beta (Abeta) and oxidative damage are critical pathological mechanisms for the development of Alzheimer’s disease. Choi et al. previously found that 4-O-methylhonokiol, extracted from *Magnolia officinalis*, improved memory dysfunction in Abeta-injected mutant mice apparently through the reduction of accumulated Abeta. Essentially this experiment was repeated, memory improved, Abeta1-42 accumulation reduced, oxidative enzymes reduced, such that 4-O-methylhonkiol is a potential candidate for delaying the onset of Alzheimer’s disease [123].

Another foodstuff that may provide some benefit to Alzheimer’s disease subjects is coconut oil from coconut (*Cocos nucifera*) which is no longer wild and is a member of the perennial monocot family, Acecaceae, but cultivated. The oil is rich in medium chain fatty acids, easily absorbed and taken up by the liver, converted to ketones, which are a rich source of energy for those struggling with memory impairment such as Alzheimer’s disease and may qualify as a functional food [124]. It has also been reported that chronic cholestasis is low in antioxidants [125].

### 3.2. Renoprotective Dietary Antioxidants

Functional foods require a specific definition and food effects may vary in relation to disease e.g., Fanti et al. have noted in end-stage-renal disease that blood isoflavonoids are accumulated in line with dietary soya intake which is higher than healthy subjects with preserved kidney function, but unconjugated and sulfated levels are comparable with healthy subjects [126]. In the Women’s Health Initiative Program of 96,196 women, when followed up, 240 had renal cell carcinoma which was inversely proportional to lycopene (assessed from a food frequency questionnaire) [127]. In another example, of impaired renal function, carboxyethyl-hydroxychramans showed an increase in plasma levels from hemodialysis subjects compared with healthy controls but it would seem that the antioxidant status does not appear to change. Clearly more research is needed, especially on the safety of dietary antioxidants [128].

### 3.3. Digestive System and Dietary Antioxidants

Dietary antioxidants such as carotenoids, flavonoids, vitamin E, and ascorbic acid present in many fruits and vegetables positively influence DNA repair mechanisms (e.g., possibly probiotics, and the antioxidant selenium [129]) such mutagenesis brought about by potential mutagens in N-nitroso compounds, fungal toxins, cooking bi-products [130]: postprandial stress caused by the latter may be ameliorated by choice of cooking oil used for frying [131]. These studies support the potential importance of dietary supplementation demonstrated by others e.g., the early study by Mongel et al. [132].

### 3.4. Cardiovascular Disease and Dietary Antioxidants

Fiber is an important dietary component that may reduce the incidence of some cancers. For example, whole-grain cereals rich in antioxidants and fiber have been reported nearly twenty years ago to be protective particularly for ischemic heart disease comprising 35,000 postmenopausal women (55–69 year) based on a 127 item food frequency questionnaire [133]. Antioxidants in total and antioxidant enzymes and lipoperoxide plasma concentrations were assayed and high values of lipoperoxide related to high blood pressure in a case-control study of older person Mexicans [134]. 

Of interest is the low level of ischemic heart disease, cardiovascular disease and arteriosclerosis in Greenlander Inuits consuming marine foods; those who subsequently died in Nuuk and Ilulissat had autopsies which revealed high levels of omega-3 fatty acids in adipose tissue and the liver contained high levels of selenium [135], as to be expected came from fish and whale food [136]. In accord with many studies on subjects, vegetable (carotenoid) intake, as adjudged from a food frequency questionnaire and a prospective randomized trial of over 22,000 male physicians, aged 40–84 year, was inversely proportional to risk of coronary heart disease [137].

### 3.5. Breast Cancer and Dietary Antioxidants

In a prospective Rotterdam breast cancer study of about 3200 subjects, aged 55 year and over, followed up for a median time of 17 year, and based on a food frequency questionnaire and antioxidant estimations for selenium, flavonoids, carotenoids, vitamins E, C and A, breast cancer incidence was measured and high levels of antioxidants were associated with lower risk of this cancer [138]. Somewhat similar results were found for breast cancer patients and controls from the Cancer Institute in Chennai, India; lower antioxidants (lutein, zeaxanthin, green-yellow vegetables) being associated with more cancers [139]. Functional food designed to reduce oxidative stress were also reported for advanced breast cancer cases but the study was small and probably lacked sufficient statistical power [140]. Similar findings for breast cancer or all-cause cancer are to be found [141,142,143,144,145,146] but not exclusively so, due to possible heterogeneity of antioxidant capabilities [147]. A Russian abstract of a paper by Peresadina et al. have reported that isoflavones and selenium intake improved the clinical presentation of patients with essential hypertension, coronary heart disease and lipid metabolism possibly associated with antioxidants [148].

## 4. Discussion

### 4.1. General

This overview was directed towards understanding the brain function in relation to dietary consumption mainly by the older person. It commenced with some early definitions of food, feeding, health, food antioxidants, agriculture and plant breeding evolution, the refashioning of the food supply for health, and factors specifically addressing the needs of the older person with regards to food color, aroma, flavor and how the brain worked to ensure dietary sufficiency. The benefits of functional foods [149] were discussed (as a prelude to food antioxidants in more detail), followed by herbal antioxidants including spices and aromatic herbs. We have also provided a brief flavor (no pun intended) of the immense field of antioxidants related to liver, kidney, digestive system, cardiovascular diseases and cancer.

### 4.2. Health Promotion

It was felt that this valuable up-to-date material was lacking an important element of health promotion [150,151,152], such as obesity [153,154], including exercise [153,155], the latter being outside the scope of this overview. Although practitioners would appreciate these potential brain-related advances, they may say this is “Much ado about nothing” [156] “we want some guidance to help the older person now.”, and a brief discourse, previously reviewed as social therapy [157], has been built into the discussion herein and in the case of Japan, to convert a negative social contribution to a positive one, a role model for the rest of the world to follow, possibly [158].

Advice on healthy eating, based on epidemiological evidence may be country dependent seeking to implement the frequency and patterns of servings. Thus, following the implementation by Sweden of a health food promotion program, called matpyramid [159], the US-Department of Agriculture suggested the Food Guide Pyramid, used for example by groups in the Republic of Ireland but who show considerable variation- those who are more disadvantaged having more health problems [160]. This is now followed by “My plate” (containing 30% grain, 40% vegetables, 10% fruit and 20% protein) [161] an easier system for people to follow. 

However, inequalities are associated with the older person on a whole raft of factors analyzed in different ways e.g., meta-analysis [162], implicit in food security [163], including social exclusion [164,165] couched in a human ecological framework [166] with its interlinked micro-, meso- and macro-components and life-course-chronosystem (implicit in chronobiology which has a wider remit well beyond this overview) where inaccessibility to food retailers and low income levels are key determinants of dietary quality and health inequality especially in the older person [167]. 

There are many such dietary schemes e.g., the Mediterranean diet, and the National Nutrition and Health Program of France, each with its difficulties of assessing adherence, including those for primary care settings [168], and delay of the onset of dementias [169]. The study of adherence to the Chinese Food Pagoda project of over 130,000 men and women aged 40–75 year who were followed up for a mean time >6 years based in Shanghai showed that a higher score of adherence was associated with lower risks of cardiometabolic diseases, cancer, and diabetes, particularly the former in men: the adherence to the following food were vegetables, fruit, legumes, fish, and eggs; but not grains, dairy, meat, fat, and salt [170].

An active area of research is dietary pattern analysis [171] and cognitive function which could be built into the follow-up of national schemes and information on dementias including Alzheimer’s disease [172]. In collaborative enterprises, industry could improve the dietary status of the older person, subject to acceptance [173]; the food industry and retailers could design better meals [174]; the food industry could like pharmaceutical products, recommend some control on healthy eating of these bioactive products [175,176]; the software manufacturer could design food apps to count dietary patterns of intake, consumption of functional foods, allergies, calories diary, accessibility, etc., [97]. Of course, this means conceptually a new generation of the older person with changing life-course [177] capable of being influenced by, and responding to, mass media [178] authoritative information on dietary advice and health matters. Labeling would certainly be valuable if print on labels is large enough or the use of symbols, such as the Healthier Choice Symbol Program of Singapore [179], was visible.

## 5. Conclusions

This overview has addressed some key brain-functions associated with dietary choices and the influence of foods on cardiovascular diseases and neurodegenerative disorders like dementias and Alzheimer’s disease. We have mentioned food inequalities within a social context in the older person. Further work is needed for coalescing the strands for progress and to address the wellbeing and dietary needs of the future generation of an older person population.
